# Fetal Fraction of Cell‐Free DNA in the Prediction of Adverse Pregnancy Outcomes: A Nationwide Retrospective Cohort Study

**DOI:** 10.1111/1471-0528.17978

**Published:** 2024-10-02

**Authors:** Ellis C. Becking, Mireille N. Bekker, Jens Henrichs, Caroline J. Bax, Erik A. Sistermans, Lidewij Henneman, Peter G. Scheffer, Ewoud Schuit

**Affiliations:** ^1^ Department of Obstetrics, Wilhelmina Children's Hospital University Medical Center Utrecht, Utrecht University Utrecht The Netherlands; ^2^ Department of Midwifery Science Amsterdam UMC, Location Vrije Universiteit Amsterdam The Netherlands; ^3^ Department of Obstetrics Amsterdam UMC, University of Amsterdam Amsterdam The Netherlands; ^4^ Amsterdam Reproduction and Development Research Institute Amsterdam UMC Amsterdam The Netherlands; ^5^ Department of Human Genetics Amsterdam UMC, Location Vrije Universiteit Amsterdam The Netherlands; ^6^ Julius Center for Health Sciences and Primary Care University Medical Center Utrecht, Utrecht University Utrecht The Netherlands

**Keywords:** adverse pregnancy outcomes, fetal fraction, non‐invasive prenatal testing, prediction

## Abstract

**Objective:**

To assess the added value of fetal fraction of cell‐free DNA in the maternal circulation in the prediction of adverse pregnancy outcomes.

**Design:**

Retrospective cohort study.

**Setting:**

Nationwide implementation study on non‐invasive prenatal testing (NIPT; TRIDENT‐2 study).

**Population:**

Pregnant women in the Netherlands opting for NIPT between June 2018 and June 2019.

**Methods:**

Two logistic regression prediction models were constructed for each adverse pregnancy outcome. The first model (base model) included prognostic clinical parameters that were selected from existing first‐trimester prediction models for adverse pregnancy outcomes. The second model (fetal fraction model) included fetal fraction as a predictor on top of the prognostic clinical parameters included in the base model. The added prognostic value of fetal fraction was assessed by comparing the base and fetal fraction models in terms of goodness of fit and predictive performance.

**Main Outcome Measures:**

Likelihood ratio test (LRT), area under the curve (AUC) and Integrated Discrimination Improvement (IDI) index.

**Results:**

The study cohort consisted of 56 110 pregnancies. The incidence of adverse pregnancy outcomes was 5.7% for hypertensive disorders of pregnancy (HDP; *n* = 3207), 10.2% for birthweight < p10 (*n* = 5726), 3.2% for birthweight < p2.3 (*n* = 1796), 3.4% for spontaneous preterm birth (sPTB; *n* = 1891), 3.4% for diabetes (*n* = 1902) and 1.3% for congenital anomalies (*n* = 741). Adding fetal fraction to the base model improved model fit for HDP, birthweight < p10, birthweight < p2.3, all sPTB, and diabetes, but not for congenital anomalies (LRT *p* < 0.05). For HDP, the AUC improved from 0.67 to 0.68 by adding fetal fraction to the base model (*p* = 0.14) with an IDI of 0.0018 (*p* < 0.0001). For birthweight < p10, the AUC improved from 0.65 to 0.66 (*p* < 0.0001) with an IDI of 0.0023 (*p* < 0.0001). For birthweight < p2.3, the AUC improved from 0.67 to 0.69 (*p* < 0.0001) with an IDI of 0.0011 (*p* < 0.0001). For all sPTB, the AUC was similar for both models (AUC 0.63, *p* = 0.021) with an IDI of 0.00028 (*p* = 0.0023). For diabetes, the AUC was similar (AUC 0.72, *p* = 0.35) with an IDI of 0.00055 (*p* = 0.00015).

**Conclusions:**

Fetal fraction has statistically significant but limited prognostic value in the prediction of adverse pregnancy outcomes in addition to known prognostic clinical parameters.

## 1 Introduction

Approximately 10% of pregnancies are complicated by adverse pregnancy outcomes, such as hypertensive disorders of pregnancy (HDP), small for gestational age neonates (SGA), spontaneous preterm birth (sPTB) and gestational diabetes mellitus (GDM) [1, 2, 3]. These complications are a major cause of fetal, neonatal and maternal morbidity and mortality worldwide [1, 2, 3, 4]. Early identification of pregnant women at risk for adverse pregnancy outcomes is essential for allowing timely risk‐reducing measures, such as early administration of aspirin and/or increased monitoring [5]. Although many first‐trimester prediction models have been developed, the performance of these models is mostly moderate and models have often not been validated or have been poorly implemented [6]. Consequently, there is a need to optimise strategies to identify pregnancies at risk.

Fetal fraction, defined as the proportion of fetal cell‐free DNA (cfDNA) in the maternal circulation, is routinely measured as a quality parameter in non‐invasive prenatal testing (NIPT). Since fetal cfDNA originates from the placenta, fetal fraction could reflect placental health and maternal pregnancy adaptation. In a nationwide retrospective cohort study of women opting for NIPT for fetal aneuploidies, we recently showed that a low fetal fraction in the first trimester of pregnancy is associated with an increased risk of developing adverse pregnancy outcomes including HDP, SGA, sPTB and diabetes [7]. The results of this study imply that fetal fraction has the potential to be used as a biomarker in obstetric care to improve first‐trimester prediction of adverse pregnancy outcomes. The prognostic value of fetal fraction as a biomarker for the prediction of adverse pregnancy outcomes is, however, not yet well established. Therefore, having identified the associations between fetal fraction and adverse pregnancy outcome, the next step is to assess the prognostic value of fetal fraction in addition to clinical characteristics. In this follow‐up study, we assessed the added prognostic value of the fetal fraction in the prediction of adverse pregnancy outcomes on top of known prognostic clinical parameters from first‐trimester prediction models [8].

## 2 Methods

### 2.1 Study Design and Population

This study was a follow‐up study of a nationwide retrospective cohort study that was performed to assess the association between fetal fraction and adverse pregnancy outcomes [7]. Details of this study, including inclusion criteria and laboratory analyses, have been described elsewhere [7]. The study cohort consisted of pregnant women with singleton pregnancies who had opted for NIPT between June 2018 and June 2019 and provided consent for the use of their data within the Dutch nationwide implementation study on NIPT (Trial by Dutch Laboratories for Evaluation of Non‐Invasive Prenatal Testing [TRIDENT]‐2) [9]. The TRIDENT‐study data were linked to the Dutch prenatal registry for prenatal screening data and the Dutch perinatal registry of pregnancy outcomes, resulting in a linked dataset of 56 110 pregnancies [10, 11].

### 2.2 Definition of Adverse Pregnancy Outcomes

Adverse pregnancy outcomes included HDP, birthweight < p10 and < p2.3, sPTB, diabetes and congenital anomalies. HDP was a combined outcome of pregnancy‐induced hypertension (PIH), preeclampsia (PE) and/or HELLP syndrome. The ISSHP classification was used to define PIH, PE and HELLP syndrome [12]. The Hoftiezer birthweight curve was used to define a birthweight < p10 and < p2.3 [13]. The definition of sPTB was a spontaneous birth between 24 and 37 weeks of gestation (GA) and divided in three categories: moderate to late (32–37 weeks GA), very preterm (28–32 weeks GA) and extremely preterm (24–28 weeks GA) [3]. Diabetes was defined as a combination of GDM (established by a 75 g 2‐h oral glucose tolerance test between 24 and 28 weeks GA [14]) and pre‐existing diabetes mellitus. The guidelines of the European Surveillance of Congenital anomalies (EUROCAT) were used to classify major fetal congenital anomalies [15].

### 2.3 Fetal Fraction

Fetal fraction was defined as the proportion (%) of fetal cfDNA in relation to the total cfDNA (maternal and fetal) in the maternal plasma and was analysed as a continuous variable. Fetal fraction was estimated with the VeriSeq v1 NIPT Assay Software. Details on laboratory assessment have been described elsewhere [7].

### 2.4 Identification of Prognostic Clinical Parameters From First Trimester Prediction Models

A systematic literature search was performed to identify first‐trimester prediction models for all of the included adverse pregnancy outcomes [8]. Because our linked dataset only included maternal characteristics and clinical parameters, prognostic clinical parameters were selected from these models based on availability in the linked dataset. An overview of the parameters included in the prediction models and exclusions by outcome is provided in Table S1. The prognostic parameters differed by outcome and included body mass index (BMI, kg/m^2^) at time of NIPT, maternal age at time of NIPT (in years), ethnicity (white/other), gravidity (number of previous conceptions), parity (number of previous pregnancies beyond 16 weeks of gestation), smoking (yes/no), method of conception (spontaneous/assisted), socio economic status score based on postal code area and conditions of obstetric history, including previous PE, previous preterm birth, previous birthweight < p10 and previous miscarriage.

### 2.5 Statistical Analysis

#### 2.5.1 Missing Data and Multiple Imputation

The overall proportion of missing values was 2.9% and the mean proportion of missing data across participant characteristics and outcomes was 2.9% (standard deviation 6.6%; Table S2). Figure S1 presents the missingness patterns of the database and shows a multivariate missingness pattern. To evaluate whether missing data in the database were missing completely at random (MCAR), an MCAR table was created in which characteristics of pregnant women without missing data were compared to pregnant women with missing data in at least one variable (Table S3). Because statistically significant differences were found between pregnant women with at least one missing value versus no missing values, meaning the absence of data was related to characteristics of the pregnant women, data were considered not MCAR. Since a complete case analysis in such a situation can result in biased estimates, missing data in the dataset were imputed using multiple imputation [16]. All included clinical outcomes and prognostic clinical parameters, including the fetal fraction, were used in the imputation model and 20 imputations were performed using predictive mean matching for continuous variables and logistic regression imputation for binary variables. Imputation was performed using the MICE package in R (version 3.16.0).

#### 2.5.2 Testing for Non‐Linearity

Associations between continuous predictors and outcomes were assessed for potential non‐linearity using restricted cubic spline plots and multiple fractional polynomials. When adding a transformed version of a continuous predictor to the model instead of the non‐transformed version significantly improved model fit, the predictor was transformed using restricted cubic splines or a transformation suggested by the multiple fractional polynomials function. If adding additional knots in the restricted cubic spline did not significantly improve model fit, the simplest model was chosen. Results of the multivariable logistic regression analysis, including potential transformations of continuous predictors, are presented in Table S4. Some deviations from linearity were found for fetal fraction for the outcomes HDP, birthweight < p10 and birthweight < p2.3.

#### 2.5.3 Determining the Added Prognostic Value of Fetal Fraction to Clinical Parameters

To allow valid assessment of the added prognostic value of the fetal fraction, we first fitted two multivariable logistic regression models for each outcome: a base model that included the aforementioned clinical parameters (see Table S1 for a detailed overview of parameters included per outcome) and a fetal fraction model that additionally included the fetal fraction as a predictor [17].

A hierarchical stepwise method was applied to assess the added prognostic value of the fetal fraction. First, a likelihood ratio test (LRT) was performed to compare the extent to which both models could describe the underlying data. The LRT was used as a golden standard test, meaning that if no statistically significant result was found (*p* > 0.05), it was assumed that the fetal fraction did not add prognostic value for that outcome [18].

Second, to quantify to what extent the fetal fraction adds prognostic value in case of a statistically significant LRT (*p* < 0.05), several predictive performance measures were calculated. The discriminative performance was described by the area under the curve (AUC) for both models and the difference in AUC of the base model versus the fetal fraction model was calculated and tested [19]. The AUC represents the ability of the model to discriminate between pregnant women that did and did not develop the outcome. An AUC of 50% is statistically equal to a random guess and an AUC of 100% represents perfect prediction for all participants. To formally test if this difference was statistically significant (*p* < 0.05), the Hanley McNeil method was used [20]. Furthermore, risk classification was assessed by the Integrated Discrimination Improvement (IDI) index, in which a larger IDI index indicates increased estimated risks for women with the outcome and decreased estimated risks for women without the outcome when using the fetal fraction model compared to the base model [21]. Additionally, the distribution in predicted risks of the outcomes was visualised by plotting these risks for the base and fetal fraction model, and quantified by calculating the variance. Higher variance (a greater variety in predicted risks) is desirable as it indicates the model could potentially better distinguish women based on their predicted risks. The fraction of new predictive information based on variance, that is, the proportion of total predictive information that was added to the model by the fetal fraction based on variance, was calculated by one minus the ratio of the variance in the base model to the variance in the fetal fraction model [22]. Finally, to visualise the change in predicted risks by adding the fetal fraction to the model, we plotted the predicted risks for each participant before (*x*‐axis) and after (*y*‐axis) adding the fetal fraction to the model. Here, more deviation from the diagonal, that is, more vertical variance, indicates the fetal fraction helps to distinguish between two women that had the same predicted risk with the base model.

All analyses were performed in the 20 imputed dataset separately and results were pooled using Rubin's rules [23]. All statistical analyses were performed in R version 4.0.3 (The R Foundation for Statistical Computing) [24].

## 3 Results

Baseline characteristics of the study cohort were previously published (Table S2) [7]. Median maternal age was 31 years (interquartile range 29–34 years) and median fetal fraction was 8% (interquartile range 6%–11%). The prevalence of adverse pregnancy outcomes was 5.7% for HDP, 10.2% for birthweight < p10, 3.2% for birthweight < p2.3, 3.4% for all sPTB, 3.4% for diabetes and 1.3% for congenital anomalies.

Measures that indicate the added prognostic value of the fetal fraction are displayed in Table 1.

**TABLE 1:** Added prognostic value of the fetal fraction in non‐invasive prenatal testing.

Pregnancy complication (*n* = 54 711)	Likelihood ratio test (LRT)			Area under the curve (AUC)				Integrated discrimination improvement (IDI) index		Fraction of new predictive information based on variance		
	Residual deviance base model	Residual deviance fetal fraction model	LRT *p* value	Base model (95% CI)	Fetal fraction model (95% CI)	Absolute change in AUC	*p*	IDI (95% CI)	*p*	Variance base model	Variance fetal fraction model	Fraction of new information
Hypertensive disorders of pregnancy	23 245	23 165	< 0.0001	0.67 (0.66–0.68)	0.68 (0.67–0.69)	0.01	0.14	0.0018 (0.0013–0.0024)	< 0.0001	0.00135	0.00143	5.4%
Birthweight < p10	31 134	31 037	< 0.0001	0.65 (0.65–0.66)	0.66 (0.65–0.67)	0.01	< 0.0001	0.0023 (0.0017–0.0028)	< 0.0001	0.00211	0.00229	7.5%
Birthweight < p2.3	10 341	10 300	< 0.0001	0.67 (0.66–0.69)	0.69 (0.67–0.71)	0.02	< 0.0001	0.0011 (0.00065–0.0016)	< 0.0001	0.000120	0.000222	10.1%
All sPTB (24–37 weeks)	16 011	16 000	0.0010	0.63 (0.61–0.64)	0.63 (0.62–0.64)	0.003	0.021	0.00028 (0.00010–0.00046)	0.0023	0.000332	0.000340	2.3%
Moderate to late (sPTB 32–37 weeks)	14 566	14 554	0.00055	0.63 (0.61–0.64)	0.63 (0.62–0.64)	0.002	0.061	0.00030 (0.00011–0.00050)	0.0023	0.000270	0.000278	2.9%
Very sPTB (28–32 weeks)^a^	1909	1908	0.42	n/a	n/a	n/a	n/a	n/a	n/a	n/a	n/a	n/a
Extremely sPTB (24–28 weeks)^a^	1111	1111	0.56	n/a	n/a	n/a	n/a	n/a	n/a	n/a	n/a	n/a
Diabetes^b^	15 463	15 441	< 0.001	0.72 (0.70–0.73)	0.72 (0.70–0.73)	−0.001	0.35	0.00055 (0.00026–0.00083)	0.00015	0.00111	0.00110	−0.5%
Congenital anomalies^a^, ^c^	7864	7862	0.11	n/a	n/a	n/a	n/a	n/a	n/a	n/a	n/a	n/a

*Note:* An overview of the variables used in the multivariable analysis is provided in Table S1. Results of all model parameters of the multivariable logistic regression analyses are available in Table S4. Only pregnancies with a gestational age at delivery ≥ 24 weeks were analysed, except if mentioned otherwise.

Abbreviations: n/a, not applicable; sPTB, spontaneous preterm birth.

^a^ No added prognostic value was found based on the LRT. Measures of predictive performance are thus not displayed in the table.

^b^ Including both pre‐existing diabetes mellitus and gestational diabetes mellitus.

^c^ Excluding all pregnancies with confirmed fetal trisomy 21, trisomy 13, or trisomy 18.

### 3.1 Likelihood Ratio Test

Based on the LRT, fetal fraction significantly improved model fit (*p* < 0.05) for HDP, birthweight < p10, birthweight < p2.3, total sPTB, moderate to late sPTB and diabetes. Model fit was not improved after addition of the fetal fraction (LRT *p* > 0.05) for extremely sPTB, very sPTB and congenital anomalies based on the LRT, indicating fetal fraction will have no added prognostic value in the prediction of these outcomes.

**FIGURE 1:**
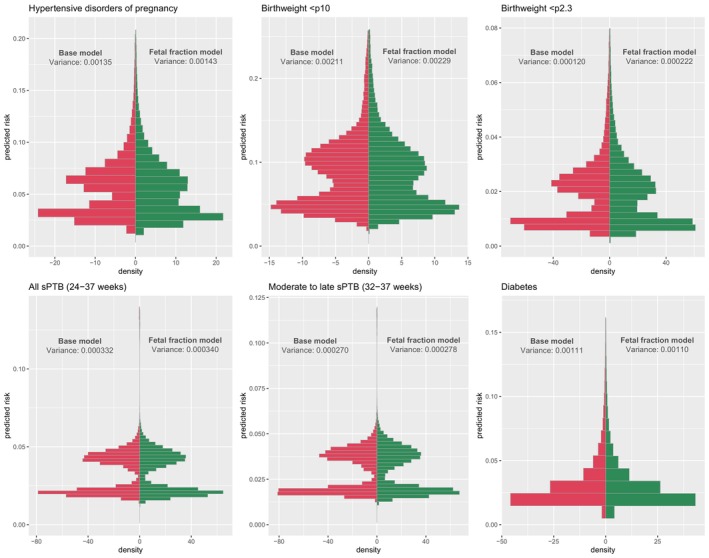
Distribution of predicted risks and variance by the base model and the extended model with fetal fraction included (fetal fraction model). sPTB; spontaneous preterm birth. The left side of the histograms (red) represent the distribution of predicted risks of the outcome by the base model. The right side of the histograms (green) represent the distribution of predicted risks of the outcome by the extended model with the fetal fraction included. A wider distribution of predicted risks and increased variance are an indication of better discrimination of a model, because the model could potentially better distinguish women with and without the outcome based on their predicted risk. The highest 1% of risk predictions are removed from this figure to improve legibility.

The quantity of prognostic value that fetal fraction added varied by type of outcome assessed. In case of a statistically significant LRT, the measures of predictive performance are displayed by outcome in Table 1. The distribution in predicted risks of the outcome by the base model and the fetal fraction model including the variance are graphically presented in Figure 1. The change in predicted risks of the outcome by the fetal fraction model versus the base model is displayed graphically in Figure S2.

### 3.2 Hypertensive Disorders of Pregnancy

For HDP, the AUC of the base model versus the fetal fraction model was 0.67 [95% CI; 0.66–0.68] versus 0.68 [95% CI; 0.67–0.69], respectively, with a *p* value for the difference in AUC of 0.14. The IDI index was 0.0018 [95% CI; 0.0013–0.0024; *p* < 0.0001], indicating statistically significant improvement in discriminatory power when fetal fraction was added to the model. The variance of the base model (0.00135) increased when fetal fraction was added to the model (0.00143) and the distribution of predicted risks widened moderately (Figure 1). The fraction of new predictive information added to the base model by the fetal fraction based on the variance was 5.4%. Moderate changes in predicted risks were seen when the fetal fraction was added to the base model (Figure S2). The change in variance and changes in predicted risks indicate that adding fetal fraction to the base model improves the ability of the model to distinguish women with a low and high risk of HDP.

### 3.3 Birthweight < p10 and < p2.3

The AUC of the base model versus the fetal fraction model for birthweight < p10 was 0.65 [95% CI; 0.65–0.66] versus 0.66 [95% CI; 0.65–0.67], respectively (*p* value of the difference in AUC < 0.0001). The IDI index was 0.0023 [95% CI; 0.0017–0.0028; *p* < 0.0001], indicating statistically significant improvement in discriminatory power when fetal fraction was added to the model. The variance of the base model (0.00211) increased when adding fetal fraction to the model (0.00229) and the distribution of predicted risks widened moderately (Figure 1). The fraction of new predictive information by adding fetal fraction to the base model was 7.5%. Considerable changes in predicted risks were seen when fetal fraction was added to the base model (Figure S2). The change in variance and changes in predicted risks indicate that adding fetal fraction to the base model improves the ability of the model to distinguish women with a low and high risk of birthweight < p10.

For birthweight < p2.3, the AUC of the base model versus the fetal fraction model increased from 0.67 [95% CI; 0.66–0.69] to 0.69 [95% CI; 0.67–0.71] (*p* value of the difference < 0.0001) with an IDI index of 0.0011 [95% CI; 0.00010–0.0016; *p* < 0.0001], indicating statistically significant improvement in discriminatory power when fetal fraction was added to the model. The variance of the base model (0.000120) increased when fetal fraction was added to the model (0.000222) and the distribution of the predicted risks widened moderately (Figure 1), resulting in 10.1% of new predictive information added by the fetal fraction. Considerable changes in predicted risks were seen when fetal fraction was added to the base model (Figure S2). The change in variance and changes in predicted risks indicate that adding fetal fraction to the base model improves the ability of the model to distinguish women with a low and high risk of birthweight < p2.3.

### 3.4 Spontaneous Preterm Birth

For the base model of all sPTB, the AUC was 0.63 [95% CI; 0.61–0.64] versus 0.63 [95% CI; 0.62–0.64] for the fetal fraction model (*p* value of the difference in AUC = 0.021). The IDI index was 0.00028 [95% CI; 0.00010–0.00046; *p* = 0.0023], indicating statistically significant improvement in discriminatory power when fetal fraction was added to the model. The variance of the base model (0.000332) increased when adding fetal fraction to the model (0.000340) and the distribution of predicted risks widened slightly (Figure 1), resulting in 2.3% new predictive information based on variance. Slight changes in predicted risks were seen when fetal fraction was added to the base model (Figure S2). The change variance and changes in predicted risks indicate that adding fetal fraction to the base model slightly improves the ability of the model to distinguish women with a high risk and low risk of all sPTB.

The AUC for moderate to late sPTB without the fetal fraction was 0.63 [95% CI; 0.61–0.64] versus 0.63 [95% CI 0.62–0.64] with the fetal fraction included in the model (*p* = 0.061). IDI index was 0.00030 [95% CI; 0.00011–0.00050; *p* = 0.0023], indicating statistically significant improvement in discriminatory power when fetal fraction was added to the model. Variance increased from 0.000270 to 0.000278 in the base model to the fetal fraction model and the distribution of predicted risks widened slightly (Figure 1). The fraction of new information based on variance was 2.9%. Slight changes in predicted risks were seen when fetal fraction was added to the base model (Figure S2). The change variance and changes in predicted risks indicate that adding fetal fraction to the base model slightly improves the ability of the model to distinguish women with a high risk and low risk of moderate to late sPTB.

### 3.5 Diabetes

For diabetes, the AUC of the base model was 0.72 [95% CI; 0.70–0.73] versus 0.72 [95% CI; 0.70–0.73] for the fetal fraction model (*p* value of the difference in AUC = 0.35). The IDI index was 0.000555 [95% CI; 0.00026–0.00083; *p* = 0.00015], indicating statistically significant improvement in discriminatory power when fetal fraction was added to the base model. The variance of the base model decreased from 0.00111 to 0.00110 in the fetal fraction model and the distribution of predicted risks were nearly similar (Figure 1), with −0.5% of predictive information retracted by the fetal fraction based on variance. Predicted risks were reasonably similar when fetal fraction was added to the base model (Figure S2). The change in variance and changes in predicted risks indicate that adding fetal fraction to the base model has no effect or even slightly deteriorates the ability of the model to distinguish women with a high risk and low risk of moderate to late sPTB.

## 4 Discussion

### 4.1 Main Findings

The aim of this nationwide retrospective cohort study of 56 110 pregnant women opting for NIPT in the Netherlands was to assess the prognostic value of fetal fraction in addition to other known prognostic clinical parameters from first trimester prediction models for adverse pregnancy outcomes. Based on the LRT, addition of the fetal fraction to the base model improved the model's ability to describe the data significantly for HDP, birthweight < p10, birthweight < p2.3, all sPTB, moderate to late sPTB and diabetes. No such improvement was found for very sPTB, extremely sPTB, or congenital anomalies. The amount of added prognostic value of fetal fraction based on the AUC, IDI index and differences in variance of predicted risks was limited, with most added prognostic value for HDP, birthweight < p10 and birthweight < p2.3. Prenatal registry for prenatal screening data and the Dutch perinatal registry of pregnancy outcomes.

### 4.2 Strengths and Limitations

A strength of this study is that we were able to assess the added prognostic value of the fetal fraction in a large nationwide cohort of 56 110 pregnant women. A limitation of our study was the presence of missing data from the national registries of prenatal screening data and pregnancy outcomes [7]. However, the proportion of missing data was low (2.9%), and multiple imputation was performed to prevent loss of power and potential bias that could result from not accounting for these missing data. A limitation of using perinatal registry data was that we were not able to add fetal fraction to the best performing developed models for adverse pregnancy because information on certain parameters, including, for instance, pre‐existing chronic hypertension, a family history of adverse pregnancy outcomes, mean arterial pressure, uterine artery pulsatility index and serum PLGF or PAPP‐A, was not available in our dataset. Whether or not fetal fraction adds prognostic value to clinically validated prediction models that include these parameters is to be established in further research.

### 4.3 Interpretation

#### 4.3.1 Translation of the Findings to Clinical Practice

In our study, we show that fetal fraction adds prognostic value to known prognostic clinical parameters in the prediction of adverse pregnancy outcomes, but also that the amount of added prognostic value is limited. The value of fetal fraction as a prognostic marker to predict adverse pregnancy outcomes is therefore questionable. However, because fetal fraction is routinely measured in almost all NIPTs as a quality parameter without additional costs or harm to patients, the fetal fraction could be used as a predictor in multivariable prediction models. Models using biochemical or ultrasound markers may have good performance, but require additional monetary or labour resources and may potentially burden pregnant women. In countries where these markers are not readily available or not routinely used, using markers such as fetal fraction could be of use. This is in line with a study by Khalil et al., that concluded that patient characteristics and cfDNA markers can potentially be used to predict PE, without the additional costs of biochemical or ultrasound markers [25].

Since we were not able to add fetal fraction to clinically validated models because data on predictors included in those models were not available. Future prospective studies should focus on adding fetal fraction to such validated and internationally used prediction models for adverse pregnancy outcomes. Additionally, if fetal fraction were to be included as a predictor in prediction models, internal and external validation of such models should be performed.

#### 4.3.2 Findings in Contrast to Other Evidence

Little previous evidence exists that aims at establishing the prognostic value of the fetal fraction in the prediction of adverse pregnancy outcomes. One study by Suzomori et al. evaluated the performance of the fetal fraction in the prediction of HDP, and found an AUC of 0.61 [95% CI; 0.55–0.66] in a model with fetal fraction, maternal age and maternal weight [26]. This is lower than the AUC for HDP reported in the current study (0.68 [95% CI; 0.67–0.69]), possibly because of the lower number of predictors included in their model. Another study by Gekas et al. [27] developed a prediction model for PE incorporating cfDNA signals, including cfDNA concentration, fetal fraction and fragment size distribution, on 100 blood samples of pregnant women. On first trimester samples with blood draws carried out at 11^+0^ until 14^+2^ weeks of gestation, the model had a 100% sensitivity and 87% specificity using a screen positive rate of 11.2%, following NICE guidelines as a threshold [28]. Khalil et al. found small changes in AUC when fetal fraction and total cfDNA were added to a model with maternal factors only in the prediction of PE (maximum AUC increase 0.01) [25]. However, in the prediction of early‐onset PE, fetal fraction in combination with total cfDNA variables had a statistically significant higher sensitivity compared to routinely available patient characteristics alone; 54%–48% (*p* < 0.05) at a screen positive rate of 15%. For preterm and term PE, the additive effect of fetal fraction was negligible.

Using fetal fraction to widen the scope of NIPT towards the prediction of adverse pregnancy outcomes fits in a broader trend of developments in the field. Other research, for instance, into using cfDNA methylation patterns in the prediction of PE shows promising results [29]. In the same way, women with a NIPT result of confined placental mosaicism have an increased risk of PE and SGA [30]. These developments suggest that in the future NIPT is likely to expand to the prediction of adverse pregnancy outcomes. Future studies could aim at further development and validation of NIPT‐based prediction models on a larger scale, making them suitable to be used in clinical practice.

Widening the scope of NIPT to the prediction of adverse pregnancy outcomes would entail a paradigm shift in the current prenatal screening landscape. The traditional aim of NIPT to increase reproductive autonomy could expand towards also a preventive aim to improve maternal and fetal health. The perspectives of pregnant women on using NIPT to predict adverse pregnancy outcomes and the ethical implications of combining test aims in one test should be examined in further research.

## 5 Conclusion

This is the first large‐scaled study investigating the potential of fetal fraction in NIPT as a biomarker to predict adverse pregnancy outcomes. We found that fetal fraction has a statistically significant but limited prognostic value in the prediction of adverse pregnancy outcomes. The limited added prognostic value of fetal fraction should be weighed against the benefits of having fetal fraction as a routinely available marker at no extra cost in NIPT in the first trimester of pregnancy. Future studies should focus on adding fetal fraction to clinically validated prediction models.

## Author Contributions

All authors have contributed to the study design and data collection. Data analysis was primarily conducted by E.C.B. and E.A.S. Data interpretation and supervision was provided by all authors. E.C.B. wrote the first draft of the manuscript. All authors reviewed and edited the subsequent versions of the manuscript.

## Ethics Statement

The TRIDENT‐2 study has been approved by the Dutch Ministry of Health, Welfare, and Sport (licence 1017420‐153371‐PG) and the Medical Ethical Committee of Amsterdam UMC, location VUMC (No. 2017.165). The Medical Ethical Committee of the VU University Medical Center (VUMC) declared that the Medical Research Involving Human Subjects Act (WMO) did not apply to this present study (No. 2020.10).

## Conflicts of Interest

The authors declare no conflicts of interest.

## Supporting information


Figure S1.

**Figure S2.** Predicted probabilities of the base model (*x*‐axis) versus the fetal fraction model (*y*‐axis) by outcome.

Table S1.

Table S2.

Table S3.

Table S4.

## Data Availability

The data that support the findings of the study are available upon reasonable request.
