# Supplementary figures and images for: Fetal Fraction of Cell‐Free DNA in the Prediction of Adverse Pregnancy Outcomes: A Nationwide Retrospective Cohort Study
Source: BJOG. 2024 Oct 2;132(3):318–25. doi: 10.1111/1471-0528.17978 (PMC11704031; doi:10.1111/1471-0528.17978)

Figure S1. Missingness pattern in the database before imputation

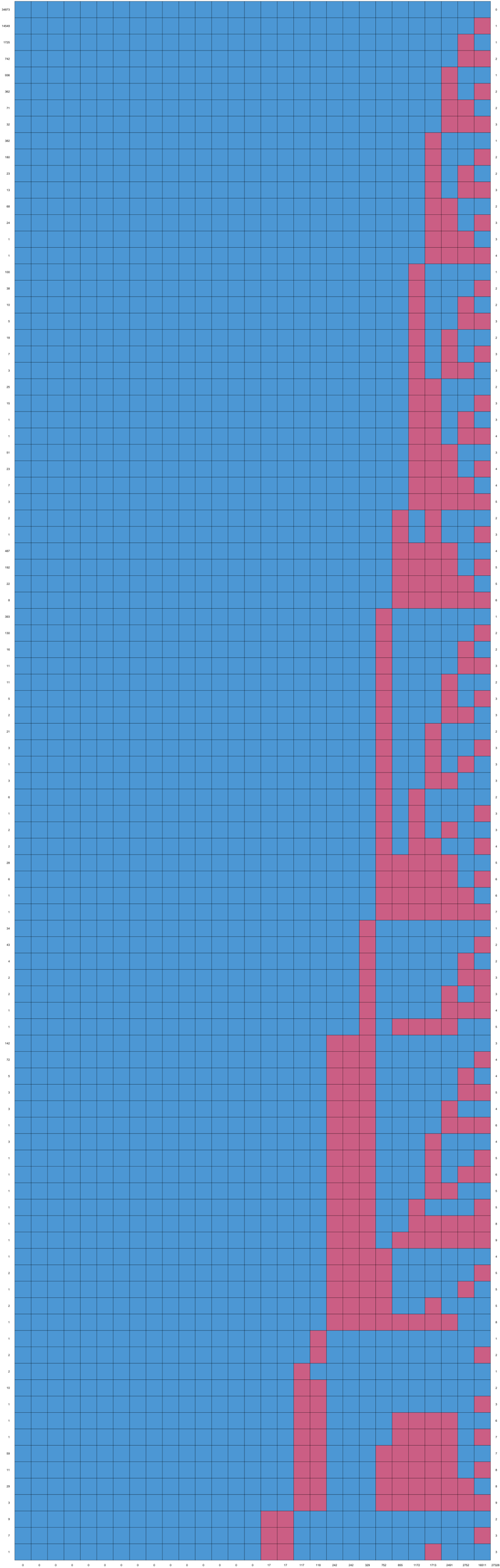

Supplement: Supplementary file 1 — Figure S1. [file BJO-132-318-s003.pdf]
