# Supplementary material for: Fetal Fraction of Cell‐Free DNA in the Prediction of Adverse Pregnancy Outcomes: A Nationwide Retrospective Cohort Study
Source: BJOG. 2024 Oct 2;132(3):318–25. doi: 10.1111/1471-0528.17978 (PMC11704031; doi:10.1111/1471-0528.17978)
Supplement: Supplementary file 2 — Figure S2. Predicted probabilities of the base model (x‐axis) versus the fetal fraction model (y‐axis) by outcome. [file BJO-132-318-s005.pdf]

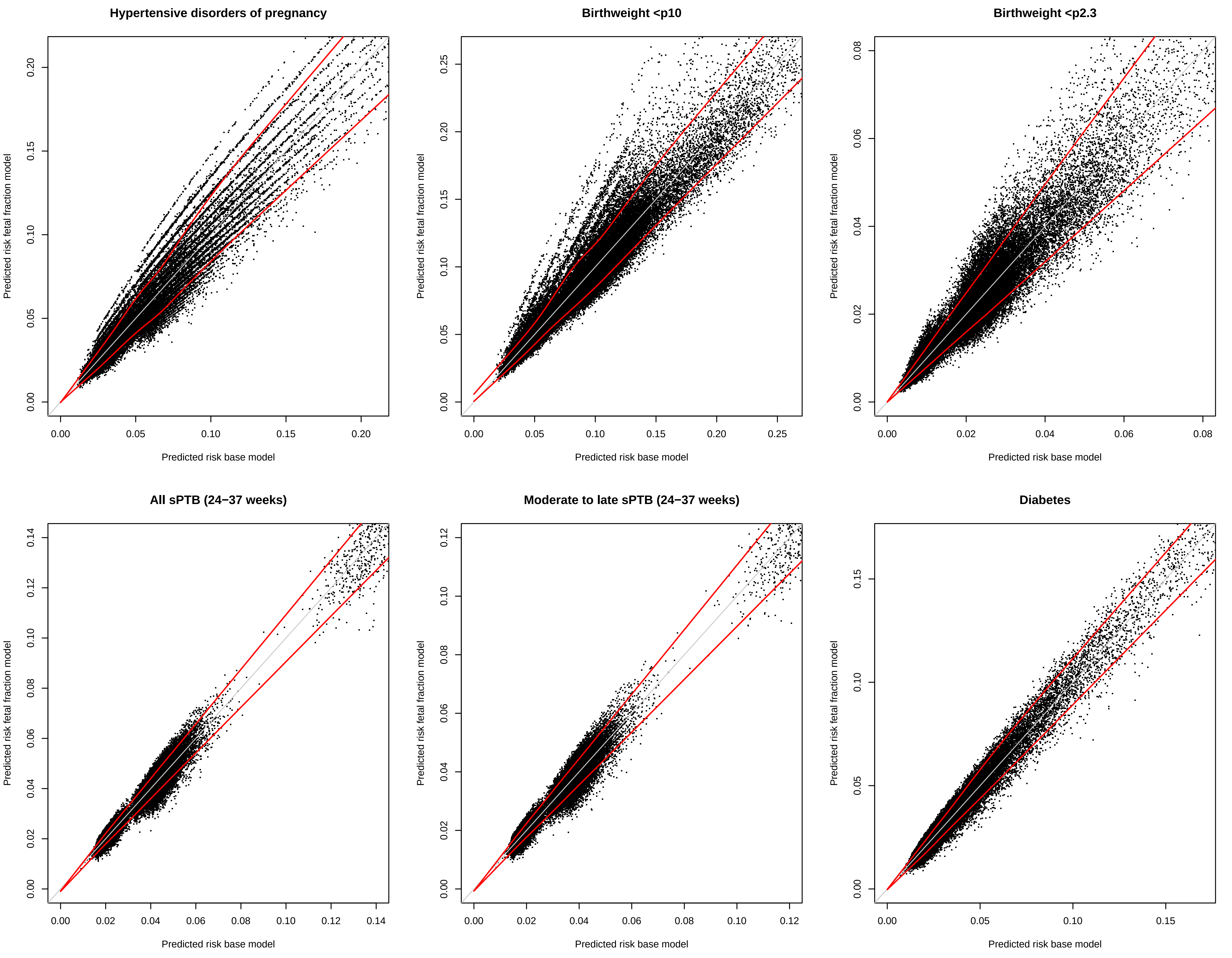
Hypertensive disorders of pregnancy
Birthweight <p10
Birthweight <p2.3
All sPTB (24−37 weeks)
Moderate to late sPTB (24−37 weeks)
Diabetes
Predicted risk fetal fraction model
Predicted risk base model
