# Supplementary material for: Fetal Fraction of Cell‐Free DNA in the Prediction of Adverse Pregnancy Outcomes: A Nationwide Retrospective Cohort Study
Source: BJOG. 2024 Oct 2;132(3):318–25. doi: 10.1111/1471-0528.17978 (PMC11704031; doi:10.1111/1471-0528.17978)
Supplement: Supplementary file 3 — Table S1. [file BJO-132-318-s004.docx]

**Table S1.** Overview of parameters used in the prediction models by outcome (indicated by an x if present in the model) and exclusions.

| **Adverse pregnancy outcomes** | **Exclusions** | **BMI** | **Maternal age** | **Ethnicity** | **Gravidity** | **Parity** | **Smoking** | **Method of conception** | **Socioeconomic status** | **Previous preeclampsia** | **Previous preterm birth** | **Previous birthweight <p10** | **Previous miscarriage** |
| --- | --- | --- | --- | --- | --- | --- | --- | --- | --- | --- | --- | --- | --- |
| Hypertensive disorders of pregnancy | GA <24 weeks | x | x | x |  | x | x | x | x | x |  |  | x |
| Birthweight < p10 | GA <24 weeks | x | x | x |  | x | x | x | x | x |  | x |  |
| Birthweight < p2.3 | GA <24 weeks | x | x | x |  | x | x | x | x | x |  | x |  |
| Diabetes^*^ | GA <24 weeks | x | x | x |  | x | x | x | x | x |  |  |  |
| All sPTB (24 - 37 weeks) | GA <24 weeks | x | x | x | x | x | x | x | x |  | x |  |  |
| Extremely sPTB  (24 - 28 weeks) | GA <24 weeks and between 28 - 37 | x | x | x | x | x | x | x | x |  | x |  |  |
| Very sPTB   (28 - 32 weeks) | GA <24 weeks and between 32 - 37 weeks | x | x | x | x | x | x | x | x |  | x |  |  |
| Moderate to late sPTB   (32 - 37 weeks) | GA < 32 weeks | x | x | x | x | x | x | x | x |  | x |  |  |
| Congenital anomalies^†^ | Cases with confirmed trisomy 21, 13, or 18 | x | x |  |  |  |  |  | x |  |  |  |  |

GA, gestational age, sPTB, spontaneous preterm birth.
