# Supplementary material for: Fetal Fraction of Cell‐Free DNA in the Prediction of Adverse Pregnancy Outcomes: A Nationwide Retrospective Cohort Study
Source: BJOG. 2024 Oct 2;132(3):318–25. doi: 10.1111/1471-0528.17978 (PMC11704031; doi:10.1111/1471-0528.17978)
Supplement: Supplementary file 4 — Table S2. [file BJO-132-318-s006.docx]

|  | Study cohort after imputation  *Median (IQR) or n (%)* | Amount of missing data  *n (% of total cohort of 56 110)* |
| --- | --- | --- |
| Baseline characteristics | (n=56,110) |  |
| Maternal age (years) | 31 (29 – 34) | 0 (0%) |
| Maternal BMI (kg/m^2^) | 23.2 (21.2 – 26.1) | 17 (0.03%) |
| Gestational age at NIPS blood draw (weeks^+ days^) | 12^+0^ (11^+4^ – 12^+5^) | 101 (0.18%) |
| Fetal fraction (%) | 8 (6 – 11) | 2752 (4.9%) |
| Ethnicity |  | 752 (1.3%) |
| White | 52 175 (93.0%) |  |
| Other | 3935 (7.0%) |  |
| Method of conception |  | 0 (0%) |
| Spontaneous | 54 733 (97.5%) |  |
| Assisted (IVF/ICSI) | 1377 (2.5%) |  |
| Smoking |  | 16 511 (29.4%) |
| Yes | 2523 (4.5%) |  |
| No | 53 587 (95.5%) |  |
| Parity |  | 117 (0.2%) |
| Nulliparous | 29 044 (51.8%) |  |
| Para 1 | 20 223 (36.0%) |  |
| Para ≥ 2 | 6843 (12.2%) |  |
| Obstetric history |  | 0 (0%) |
| Previous preeclampsia^*^ | 160/27 066 (0.6%) |  |
| Previous preterm birth^*^ | 785/27 066 (2.9%) |  |
| Previous small for gestational age^*^ | 410/27 066 (1.5%) |  |
| Previous miscarriage/abortion | 349 (0.6%) |  |
| Pregnancy outcomes |  |  |
| Gestational age at delivery (weeks) | 39^+5^ (38^+5^ - 40^+5^) | 855 (1.5%) |
| Mode of delivery |  | 2771 (4.9%) |
| Vaginal delivery | 42 505 (75.7%) |  |
| Assisted vaginal delivery (vacuum/forceps) | 4707 (8.4%) |  |
| Elective caesarean section | 4148 (7.4%) |  |
| Emergency caesarean section | 4750 (8.5%) |  |
| Birthweight (gram) | 3460 (3120 - 3785) | 1046 (1.9%) |
| Hypertensive disorders of pregnancy | 3207 (5.7%) | 0 (0%) |
| Birthweight < p10 | 5726 (10.2%) | 1713 (3.1%) |
| Birthweight < p2.3 | 1796 (3.2%) | 1713 (3.1%) |
| All sPTB (24 - 37 weeks) | 1891 (3.4%) | 1069 (1.9%) |
| Moderate to late sPTB (32 - 37 weeks) | 1675 (3.0%) |  |
| Very sPTB (28 - 32 weeks) | 140 (0.3%) |  |
| Extremely sPTB (24 - 28 weeks) | 76 (0.1%) |  |
| Diabetes^†^ | 1902 (3.4%) | 0 (0%) |
| Congenital anomalies^‡^ | 741 (1.3%) | 0 (0%) |

**Table S2. Characteristics of the study cohort**

*This table was adjusted from a previous publication by Becking et al. (AJOG, 2024)*
IVF, in vitro fertilization; ICSI, intracytoplasmic sperm injection; sPTB, spontaneous preterm birth.
^*^ Data of multiparous women only (n=27 066).
^†^ Including both pre-existing diabetes mellitus and gestational diabetes mellitus.
^¶^ Including neonatal death occurring within the first 4 completed weeks of life, Neonatal Intensive Care Unit (NICU) admission >32 weeks of GA
^‡^ Excluding pregnancies with confirmed Down, Edwards, or Patau syndrome
