# Supplementary material for: Fetal Fraction of Cell‐Free DNA in the Prediction of Adverse Pregnancy Outcomes: A Nationwide Retrospective Cohort Study
Source: BJOG. 2024 Oct 2;132(3):318–25. doi: 10.1111/1471-0528.17978 (PMC11704031; doi:10.1111/1471-0528.17978)
Supplement: Supplementary file 5 — Table S3. [file BJO-132-318-s002.docx]

**Table S3.** Characteristics of pregnant women without missing data and pregnant women with missing data in ≥1 variable.

|  | Pregnant women without missing data | Pregnant women with missing data | p-value |
| --- | --- | --- | --- |
| n | 34873 | 21237 |  |
| Fetal fraction (mean (SD)) | 8.35 (3.86) | 8.43 (3.90) | 0.028 |
| Gestational age (mean (SD)) | 277.11 (11.36) | 272.68 (26.26) | <0.001 |
| Gravidity (mean (SD)) | 2.03 (1.19) | 2.04 (1.23) | 0.334 |
| Parity (mean (SD)) | 0.64 (0.78) | 0.62 (0.78) | 0.001 |
| Maternal length (mean (SD)) | 169.48 (6.67) | 169.49 (6.90) | 0.881 |
| Maternal weight (mean (SD)) | 69.30 (13.09) | 69.85 (13.88) | <0.001 |
| Maternal age (mean (SD)) | 31.53 (4.07) | 31.79 (4.22) | <0.001 |
| Socio economic status score (mean (SD)) | 0.08 (1.14) | 0.05 (1.18) | <0.001 |
| Previous abortion/miscarriage (%) | 143 (0.4) | 206 (1.0) | <0.001 |
| Previous hypertensive disorder of pregnancy (%) | 97 (0.3) | 63 (0.3) | 0.751 |
| Previous preterm birth (%) | 407 (1.2) | 378 (1.8) | <0.001 |
| Previous SGA (%) | 253 (0.7) | 157 (0.7) | 0.893 |
| Level of urbanisation (%) |  |  | <0.001 |
| >2500 inhabitants/m^2^ | 16578 (47.5) | 10736 (51.1) |  |
| 1500-2500 inhabitants/m^2^ | 3542 (10.2) | 1921 (9.1) |  |
| 1000-1500 inhabitants/m^2^ | 2561 (7.3) | 1421 (6.8) |  |
| 500-1000 inhabitants/m^2^ | 4345 (12.5) | 2771 (13.2) |  |
| <500 inhabitants/m^2^ | 7847 (22.5) | 4146 (19.7) |  |
| Method of conception = IVF/ICSI (%) | 1051 (3.0) | 326 (1.5) | <0.001 |
| Ethnicity = white (%) | 32789 (94.0) | 18693 (91.3) | <0.001 |
| Smoking = no (%) | 33370 (95.7) | 4479 (94.8) | 0.005 |
| Deprived area of living = yes (%) | 3266 (9.4) | 2375 (11.3) | <0.001 |
| Diabetes = yes (%) | 1243 (3.6) | 659 (3.1) | 0.004 |
| Hypertensive disorders of pregnancy (%) | 2035 (5.8) | 1049 (4.9) | <0.001 |
| Preeclampsia/HELLP (%) | 133 (0.4) | 65 (0.3) | 0.166 |
| Hoftiezer percentile (mean (SD)) | 50.96 (28.61) | 50.55 (29.05) | 0.103 |
| Start of birth (%) |  |  | <0.001 |
| Spontaneous | 24693 (70.8) | 12604 (67.2) |  |
| Induced: amniotomy | 2674 (7.7) | 1590 (8.5) |  |
| Induced: prostaglandins | 786 (2.3) | 753 (4.0) |  |
| Induced: oxytocin | 558 (1.6) | 433 (2.3) |  |
| Induced: prostaglandins + oxytocin | 48 (0.1) | 38 (0.2) |  |
| Primary caesarean section | 2602 (7.5) | 1434 (7.6) |  |
| Foley catheter | 3512 (10.1) | 1894 (10.1) |  |
| Congenital anomaly = yes (%) | 398 (1.1) | 441 (2.1) | <0.001 |
| Neonatal mortality (%) |  |  | <0.001 |
| Alive | 34828 (99.9) | 19700 (92.8) |  |
| Death before birth | 0 (0.0) | 124 (0.6) |  |
| Not viable | 0 (0.0) | 1262 (5.9) |  |
| Death through birth | 0 (0.0) | 64 (0.3) |  |
| Death <24h after birth | 20 (0.1) | 57 (0.3) |  |
| Death 2^nd^-7^th^ day after birth | 12 (0.0) | 18 (0.1) |  |
| Death > 8^th^  day after birth | 13 (0.0) | 12 (0.0) |  |
| missing = TRUE (%) | 0 (0.0) | 21237 (100.0) | <0.001 |
