# Supplementary material for: Fetal Fraction of Cell‐Free DNA in the Prediction of Adverse Pregnancy Outcomes: A Nationwide Retrospective Cohort Study
Source: BJOG. 2024 Oct 2;132(3):318–25. doi: 10.1111/1471-0528.17978 (PMC11704031; doi:10.1111/1471-0528.17978)
Supplement: Supplementary file 6 — Table S4. [file BJO-132-318-s001.docx]

**Table S4.** Results of the multivariable logistic regression analysis.

| **Hypertensive disorders of pregnancy** | **B-coefficient** | **bSE** | **OR** | **CI 95% LB** | **CI 95% UB** | **p-value** |
| --- | --- | --- | --- | --- | --- | --- |
| (Intercept) | -3.54 | 0.18 | 0.03 | 0.02 | 0.04 | <0.0001 |
| (BMI/10)^1) + (BMI/10)^2 | 0.10 | 0.01 | 1.10 | 1.09 | 1.11 | <0.0001 |
| (Age/10)^2 + (Age/10)^3 | 0.00 | 0.00 | 1.00 | 1.00 | 1.00 | 0.43 |
| Ethnicity (other) | -0.50 | 0.09 | 0.60 | 0.51 | 0.72 | <0.0001 |
| (Parity+ 1)^-2 | 1.01 | 0.05 | 2.75 | 2.47 | 3.06 | <0.0001 |
| Method of conception (assisted) | 0.22 | 0.11 | 1.25 | 1.01 | 1.54 | 0.04 |
| Smoking (no) | 0.02 | 0.11 | 1.02 | 0.83 | 1.26 | 0.83 |
| Previous abortion/miscarriage | 0.17 | 0.28 | 1.18 | 0.69 | 2.04 | 0.54 |
| Previous preeclampsia | 2.29 | 0.18 | 9.87 | 6.87 | 14.17 | <0.0001 |
| Previous birthweight <p10 | 0.17 | 0.24 | 1.18 | 0.73 | 1.90 | 0.50 |
| (Socio economic status + 6.5)/10)^3 | -0.11 | 0.13 | 0.89 | 0.69 | 1.16 | 0.40 |
| (fetal fraction+1/10)^0.5 | -0.83 | 0.09 | 0.44 | 0.36 | 0.53 | <0.0001 |

| **Birthweight <p10** | **B-coefficient** | **bSE** | **OR** | **CI 95% LB** | **CI 95% UB** | **p-value** |
| --- | --- | --- | --- | --- | --- | --- |
| (Intercept) | -4.86 | 0.13 | 0.01 | 0.01 | 0.01 | <0.0001 |
| (BMI/10)^-2) + (BMI/10)^-2 * log(BMI/10) | 3.10 | 0.22 | 22.22 | 14.51 | 34.03 | <0.0001 |
| (Age/10)^3) + (Age/10)^3 * log(Age/10) | 0.00 | 0.00 | 1.00 | 1.00 | 1.00 | <0.0001 |
| Ethnicity (other) | 0.60 | 0.05 | 1.82 | 1.65 | 2.01 | <0.0001 |
| (Parity + 1)^-1 + (Parity + 1)^-0.5 | 1.03 | 0.04 | 2.81 | 2.59 | 3.04 | <0.0001 |
| Method of conception (assisted) | 0.03 | 0.09 | 1.03 | 0.86 | 1.24 | 0.76 |
| Smoking (no) | -0.72 | 0.07 | 0.49 | 0.43 | 0.56 | <0.0001 |
| Previous preeclampsia | 0.14 | 0.31 | 1.15 | 0.63 | 2.11 | 0.65 |
| Previous birthweight<p10 | 1.83 | 0.12 | 6.24 | 4.96 | 7.85 | <0.0001 |
| Socio economic status | -0.02 | 0.01 | 0.98 | 0.95 | 1.00 | 0.07 |
| log(fetal fraction+1/10) | -0.31 | 0.03 | 0.73 | 0.69 | 0.78 | <0.0001 |

| **Birthweight <p2.3** | **B-coefficient** | **bSE** | **OR** | **CI 95% LB** | **CI 95% UB** | **p-value** |
| --- | --- | --- | --- | --- | --- | --- |
| (Intercept) | -5.80 | 0.28 | 0.00 | 0.00 | 0.01 | <0.0001 |
| (BMI/10)^-2 + (BMI/10)^-2 * log(BMI/10) | 1.91 | 0.43 | 6.78 | 2.94 | 15.62 | <0.0001 |
| (Age/10)^2) + (Age/10)^2 * log(Age/10) | 0.03 | 0.00 | 1.03 | 1.02 | 1.04 | <0.0001 |
| Ethnicity (other) | 0.60 | 0.10 | 1.81 | 1.50 | 2.19 | <0.0001 |
| (Parity + 1)^-1 + (Parity + 1)^-0.5 | 1.34 | 0.09 | 3.83 | 3.23 | 4.53 | <0.0001 |
| Method of conception (assisted) | 0.09 | 0.17 | 1.09 | 0.78 | 1.53 | 0.61 |
| Smoking (no) | -0.92 | 0.11 | 0.40 | 0.32 | 0.50 | <0.0001 |
| Previous preeclampsia | 0.36 | 0.59 | 1.44 | 0.45 | 4.61 | 0.54 |
| Previous birthweight<p10 | 1.80 | 0.22 | 6.05 | 3.95 | 9.28 | <0.0001 |
| Socio economic status | -0.07 | 0.03 | 0.94 | 0.89 | 0.98 | 0.01 |
| (fetal fraction+1/10)^0.5 | -0.98 | 0.15 | 0.38 | 0.28 | 0.51 | <0.0001 |

| **All sPTB (24-37 weeks GA)** | **B-coefficient** | **bSE** | **OR** | **CI 95% LB** | **CI 95% UB** | **p-value** |
| --- | --- | --- | --- | --- | --- | --- |
| (Intercept) | -1.36 | 0.64 | 0.26 | 0.07 | 0.90 | 0.03 |
| rcs(BMI, 4) | -0.12 | 0.03 | 0.88 | 0.83 | 0.94 | <0.0001 |
| rcs(BMI, 4) | 0.70 | 0.19 | 2.02 | 1.40 | 2.91 | 0.00017 |
| rcs(BMI, 4) | -1.64 | 0.45 | 0.19 | 0.08 | 0.46 | 0.00022 |
| (Age/10)^3) + (Age/10)^3 * log(Age/10) | 0.00 | 0.00 | 1.00 | 1.00 | 1.00 | 0.027 |
| Ethnicity (other) | 0.10 | 0.09 | 1.10 | 0.93 | 1.32 | 0.27 |
| (Parity+ 1)^-2 | 0.82 | 0.11 | 2.28 | 1.85 | 2.81 | <0.0001 |
| (Gravidity)^-2 | 0.22 | 0.10 | 1.24 | 1.03 | 1.50 | 0.02 |
| Method of conception (assisted) | 0.04 | 0.15 | 1.04 | 0.79 | 1.39 | 0.77 |
| Smoking (no) | -0.16 | 0.12 | 0.85 | 0.67 | 1.07 | 0.17 |
| Previous preterm birth | 2.09 | 0.11 | 8.07 | 6.47 | 10.07 | <0.0001 |
| Socio economic status | -0.03 | 0.02 | 0.98 | 0.94 | 1.02 | 0.22 |
| Fetal fraction | -0.02 | 0.01 | 0.98 | 0.97 | 0.99 | 0.0014 |

| **Extremely sPTB (24-28 weeks GA)** | **B-coefficient** | **bSE** | **OR** | **CI 95% LB** | **CI 95% UB** | **p-value** |
| --- | --- | --- | --- | --- | --- | --- |
| (Intercept) | -6.78 | 2.08 | 0.00 | 0.00 | 0.07 | 0.0011 |
| rcs(Age, 3) | -0.06 | 0.06 | 0.94 | 0.84 | 1.06 | 0.30 |
| rcs(Age, 3) | 0.14 | 0.08 | 1.15 | 0.98 | 1.34 | 0.09 |
| BMI | 0.32 | 0.40 | 1.37 | 0.62 | 3.03 | 0.43 |
| Ethnicity (other) | 1.67 | 0.43 | 5.30 | 2.26 | 12.43 | 0.00012 |
| Gravidity | 0.14 | 0.12 | 1.15 | 0.91 | 1.44 | 0.23 |
| (Parity+ 1)^-2 | 0.00 | 0.03 | 1.00 | 0.94 | 1.06 | 0.97 |
| Method of conception (assisted) | 0.48 | 0.55 | 1.61 | 0.55 | 4.74 | 0.39 |
| Smoking (no) | 0.20 | 0.68 | 1.22 | 0.32 | 4.59 | 0.77 |
| Previous preterm birth | 2.33 | 0.54 | 10.23 | 3.58 | 29.23 | 0.000014 |
| Socio economic status | 0.05 | 0.10 | 1.05 | 0.86 | 1.28 | 0.66 |
| Fetal fraction | 0.02 | 0.03 | 1.02 | 0.95 | 1.08 | 0.63 |

| **Very sPTB (28-32 weeks GA)** | **B-coefficient** | **bSE** | **OR** | **CI 95% LB** | **CI 95% UB** | **p-value** |
| --- | --- | --- | --- | --- | --- | --- |
| (Intercept) | -6.63 | 1.09 | 0.00 | 0.00 | 0.01 | <0.0001 |
| BMI | 0.04 | 0.02 | 1.04 | 0.99 | 1.09 | 0.11 |
| Age | 0.47 | 0.29 | 1.60 | 0.92 | 2.80 | 0.10 |
| Ethnicity (other) | -0.37 | 0.21 | 0.69 | 0.46 | 1.04 | 0.08 |
| Parity | 0.26 | 0.33 | 1.30 | 0.68 | 2.49 | 0.43 |
| (Gravidity)^-2 | -0.35 | 0.63 | 0.71 | 0.20 | 2.44 | 0.58 |
| Method of conception (assisted) | -0.28 | 0.41 | 0.76 | 0.34 | 1.70 | 0.50 |
| Smoking (no) | 2.02 | 0.39 | 7.57 | 3.53 | 16.25 | <0.0001 |
| Previous preterm birth | -0.01 | 0.08 | 0.99 | 0.85 | 1.15 | 0.90 |
| Socio economic status | 0.00 | 0.02 | 1.00 | 0.96 | 1.04 | 0.89 |
| Fetal fraction | -0.02 | 0.02 | 0.98 | 0.94 | 1.03 | 0.47 |

| **Moderate to late sPTB (32-37 weeks GA)** | **B-coefficient** | **bSE** | **OR** | **CI 95% LB** | **CI 95% UB** | **p-value** |
| --- | --- | --- | --- | --- | --- | --- |
| (Intercept) | -1.32 | 0.70 | 0.27 | 0.07 | 1.06 | 0.06 |
| rcs(BMI, 4) | -0.13 | 0.03 | 0.87 | 0.82 | 0.93 | <0.0001 |
| rcs(BMI, 4) | 0.75 | 0.20 | 2.11 | 1.44 | 3.11 | 0.00014 |
| rcs(BMI, 4) | -1.74 | 0.47 | 0.18 | 0.07 | 0.44 | 0.00021 |
| Age | 0.01 | 0.01 | 1.01 | 0.99 | 1.02 | 0.25 |
| Ethnicity (other) | 0.05 | 0.10 | 1.05 | 0.87 | 1.28 | 0.59 |
| (Parity+ 1)^-2 | 0.76 | 0.11 | 2.15 | 1.72 | 2.68 | <0.0001 |
| (Gravidity)^-2 | 0.26 | 0.10 | 1.30 | 1.06 | 1.58 | 0.01 |
| Method of conception (assisted) | 0.05 | 0.15 | 1.05 | 0.78 | 1.43 | 0.73 |
| Smoking (no) | -0.17 | 0.12 | 0.85 | 0.67 | 1.08 | 0.17 |
| Previous preterm birth | 2.09 | 0.12 | 8.10 | 6.41 | 10.23 | <0.0001 |
| Socio economic status | -0.03 | 0.02 | 0.97 | 0.93 | 1.01 | 0.17 |
| Fetal fraction | -0.02 | 0.01 | 0.98 | 0.96 | 0.99 | 0.00087 |

| **Diabetes** | **B-coefficient** | **bSE** | **OR** | **CI 95% LB** | **CI 95% UB** | **p-value** |
| --- | --- | --- | --- | --- | --- | --- |
| (Intercept) | -4.59 | 0.17 | 0.01 | 0.01 | 0.01 | <0.0001 |
| rcs(gravidity, 3) | 0.15 | 0.07 | 1.17 | 1.02 | 1.33 | 0.02 |
| rcs(gravidity, 3) | -0.22 | 0.08 | 0.80 | 0.68 | 0.94 | 0.01 |
| (Age/10)^3 | 0.07 | 0.00 | 1.07 | 1.06 | 1.08 | <0.0001 |
| Ethnicity (other) | 0.01 | 0.00 | 1.01 | 1.01 | 1.02 | <0.0001 |
| Parity | 0.83 | 0.07 | 2.29 | 2.00 | 2.63 | <0.0001 |
| Method of conception (assisted) | -0.07 | 0.05 | 0.93 | 0.85 | 1.02 | 0.13 |
| Smoking (no) | 0.60 | 0.12 | 1.83 | 1.46 | 2.29 | <0.0001 |
| Previous preterm birth | -0.12 | 0.12 | 0.89 | 0.70 | 1.14 | 0.35 |
| Socio economic status | -0.21 | 0.46 | 0.81 | 0.33 | 2.01 | 0.66 |
| (BMI/10)^2 + (BMI/10)^2 * log(BMI/10) | -0.09 | 0.02 | 0.92 | 0.88 | 0.95 | <0.0001 |
| Fetal fraction | -0.03 | 0.01 | 0.97 | 0.96 | 0.98 | <0.0001 |

| **Congenital anomalies** | **B-coefficient** | **bSE** | **OR** | **CI 95% LB** | **CI 95% UB** | **p-value** |
| --- | --- | --- | --- | --- | --- | --- |
| (Intercept) | -4.79 | 0.38 | 0.01 | 0.00 | 0.02 | <0.0001 |
| Age | 0.00 | 0.01 | 1.00 | 0.98 | 1.02 | 0.83 |
| BMI | -0.01 | 0.03 | 0.99 | 0.93 | 1.06 | 0.83 |
| Socio economic status | 0.03 | 0.01 | 1.03 | 1.01 | 1.04 | 0.00094 |
| Fetal fraction | -0.02 | 0.01 | 0.98 | 0.96 | 1.00 | 0.13 |

*If a transformation was used, this is indicated in the variable name. If restricted cubic splines were used, this is indicated by rcs(variable name, number of splines). Age represents the maternal age in years. Abbreviations: sPTB: spontaneous preterm birth; GA: gestational age; bSE: standard error of beta coefficient; OR: odds ratio; CI: confidence interval; LB: lower bound; UB: upper bound*
